# Sensor Technology for Smart Homes

**DOI:** 10.3390/s20247046

**Published:** 2020-12-09

**Authors:** Juan Ye, Michael O’Grady, Oresti Banos

**Affiliations:** 1 School of Computer Science, University of St Andrews, St. Andrews KY16 9SX, UK; 2 School of Computer Science and Informatics, University College Dublin, Dublin 4, Ireland; michael.j.ogrady@ucd.ie; 3 Faculty ETSIIT, University of Granada, 18010 Granada, Spain; oresti@ugr.es

As advances in technology continue relentlessly, intriguing possibilities for smart home services have emerged. Nonetheless, significant obstacles remain before smart home and equivalent technologies reach a sufficiently high Technology Readiness Level (TRL) and thus become mainstream. The challenges that must be overcome are many; the inherent complexity of the smart home, though a constrained environment in many ways, poses many difficulties. Some difficulties are technical—the lack of robust and scalable technologies, for example. Human factors and even sociotechnical issues may drive other problems that arise. Holistic solutions for the smart home will only emerge from transdisciplinary research and collaborations.

At the time of writing, the second phase of the COVID-19 pandemic is peaking. A radical reassessment of what constitutes normalcy has resulted. Homes have increasingly become places of work and enterprise, posing many challenges for society, employers and families. Older adults and other vulnerable people have often been obliged to live in isolation for extended periods, often aggravating mental and physical health problems. Thus, going forward, it may be instructive to perhaps revisit the smart home both conceptually and phenomenologically, rather than as a manifestation of technology in the first instance.

## Summary of this Special Issue

Resilient smart homes demand the availability of reliable, fault-tolerant sensor networks. Detecting sensor failure as early as possible is essential for ensuring trustworthiness and confidence. In [1], a mechanism for sensor failure detection and subsequent sensor isolation based on association rule mining is proposed for event-driven, ambient sensors. Promising results have emerged in the cases of unlabelled datasets and unknown sensor topologies.

In [2], the importance of occupancy management for smart homes, but without compromising privacy, is considered. An innovative method based on the prediction of CO_2_ concentration is employed. An Artificial Neural Network, augmented with a wavelet transformation for additive noise cancelling, is harnessed. Initial results from this indirect monitoring of human daily living activities are impressive, showing significant potential for addressing the omnipresent ethical and privacy issues in smart home research.

A complementary approach, again using CO_2_ as a predictor of occupancy, is described in [3]. In contrast, this approach focuses on occupancy detection and the number of occupants. A Fibre Bragg Grating (FBG) sensor is central to the proposed approach. Their small form factor makes their deployment easy and cost-effective, and thus an apt solution for smart homes.

Fall detection systems have been the subject of intense research over the last decade. The advantages of robust systems for the automated detection of falls is desirable for all the obvious reasons; however, as yet, none are on the market. The impossibility of generating real-world datasets from falls by older adults in real-world settings significantly exacerbates the difficulties in creating effective prediction systems. Thus, the eHomeSeniors Dataset [4] has been expressly constructed in collaboration with a physiotherapist, younger people, and performing artists, using privacy-preserving infrared sensors. This dataset is in the public domain.

Nonintrusive Appliance Load Monitoring (NIALM) offers a transparent approach to monitoring energy usage in the smart home at the device-level. Such functionality is indispensable for delivering services that advise on sustainable and cost-effective energy usage. Tracking appliance usage is also a useful proxy for occupancy and behaviour monitoring. Delivering robust, scalable NIALM systems for smart homes is an attractive proposition but one fraught with difficulty at present. In [5], the authors describe algorithms for signal processing within a NIALM context and present initial findings.

Potential occupants of the smart home will be the ultimate judges of the home’s smartness. Multi-agent systems offer an intriguing solution for managing the various devices and catering to the occupant’s needs. In [6], the authors describe a gamification approach, built on the multi-agent paradigm, for assigning and distributing tasks amongst occupants.

A key hindrance to the adaptation of smart home technologies concerns the need for a dedicated sensor infrastructure and the challenges that result from the installation and management of these infrastructures. Device-free activity-recognition systems offer a desirable alternative, minimizing the need for infrastructure, eliminating the need for Line-of-Sight (LoS) and maximining privacy issues. In [7], the authors explore the impact of the environment on the efficacy of Wi-Fi, resulting in insights as to how smart homes might be configured to harness Wi-Fi for device-free activity recognition.

One group of occupants for whom the promise of smart homes may be positively life-changing is people with severe disabilities. A novel assistive technology system based on eye-tracking is proposed in [8]. Here, usability assessments, by those both with and without disability, of the proposed system are positive.

To conclude: a scientific literature review of technologies used for activity recognition in smart homes and ambient assisted living scenarios is presented in [9].
